# Co-option of the bZIP transcription factor Vrille as the activator of *Doublesex1* in environmental sex determination of the crustacean *Daphnia magna*

**DOI:** 10.1371/journal.pgen.1006953

**Published:** 2017-11-02

**Authors:** Nur Syafiqah Mohamad Ishak, Quang Dang Nong, Tomoaki Matsuura, Yasuhiko Kato, Hajime Watanabe

**Affiliations:** 1 Department of Biotechnology, Graduate School of Engineering, Osaka University, Suita, Osaka, Japan; 2 Biotechnology Global Human Resource Development Program, Division of Advanced Science and Biotechnology, Department of Biotechnology, Graduate School of Engineering, Osaka University, Suita, Osaka, Japan; 3 Frontier Research Base of Global Young Researchers, Graduate School of Engineering, Osaka University, Suita, Japan; New York University, UNITED STATES

## Abstract

Divergence of upstream regulatory pathways of the transcription factor *Doublesex* (*Dsx*) serves as a basis for evolution of sex-determining mechanisms in animals. However, little is known about the regulation of *Dsx* in environmental sex determination. In the crustacean *Daphnia magna*, environmental sex determination is implemented by male-specific expression of the *Dsx* ortholog, *Dsx1*. Transcriptional regulation of *Dsx1* comprises at least three phases during embryogenesis: non-sex-specific initiation, male-specific up-regulation, and its maintenance. Herein, we demonstrate that the male-specific up-regulation is controlled by the bZIP transcription factor, *Vrille* (*Vri*), an ortholog of the circadian clock genes—*Drosophila Vri* and mammalian *E4BP4/NFIL3*. Sequence analysis of the *Dsx1* promoter/enhancer revealed a conserved element among two *Daphnia* species (*D*. *magna* and *D*. *pulex*), which contains a potential enhancer harboring a consensus Vri binding site overlapped with a consensus Dsx binding site. Besides non-sex-specific expression of *Vri* in late embryos, we found male-specific expression in early gastrula before the *Dsx1* up-regulation phase begins. Knockdown of *Vri* in male embryos showed reduction of *Dsx1* expression. In addition, transient overexpression of *Vri* in early female embryos up-regulated the expression of *Dsx1* and induced male-specific trait. Targeted mutagenesis using CRISPR/Cas9 disrupted the enhancer on genome in males, which led to the reduction of *Dsx1* expression. These results indicate that *Vri* was co-opted as a transcriptional activator of *Dsx1* in environmental sex determination of *D*. *magna*. The data suggests the remarkably plastic nature of gene regulatory network in sex determination.

## Author summary

Sex is widespread for reproduction of offspring in the animal kingdom. In the sex determination process, through interactions of several genes in a hierarchical manner, an initial cue leads to sex-specific expression of the major effector of sexual differentiation, *Doublesex* (*Dsx*). Although how genetic factors on sex chromosomes control *Dsx* expression has been extensively studied in model organisms such as mouse, fruit fly, and nematodes, little is known about dependence of *Dsx* regulation on environmental signals. We used the crustacean, *Daphnia magna*, owing to its advantages for analyzing environmental sex determination: 1) fully sequenced genome, 2) recent advancement of genome engineering and 3) artificial control of sex by juvenile hormone treatment. We found that early male embryos transiently express the bZIP transcription factor, *Vrille* (*Vri*), known to be a circadian regulator, before male-specific *Dsx1* activation begins. Disruption of a potential Vri-binding site in the *Dsx1* regulatory region, and gain- and loss-of-function analyses revealed that *Vri* regulates male-specific *Dsx1* activation in *Daphnia*. We infer that a novel gene can be co-opted as a regulator of *Dsx* in environmental sex-determining pathway. Our results would expand our understanding about the diversity and evolution of the sex-determining pathways in animals.

## Introduction

The diversity and evolution of sex-determining pathways among animals are fundamental issues in developmental and evolutionary biology. The primary cues to trigger sexual development have been varied across evolution [1,2], and can be broadly divided into two categories: a strict genetic cue or merely an environmental signal [3]. There are numerous studies about genetic sex determination (GSD) mechanisms from various model organisms, including the mouse, nematode, and fruit fly. These studies have shown that, through interactions of several genes in a hierarchical manner, initial cues finally lead to sex-specific expression of the major effector of sexual differentiation, a DM-domain gene that encodes a transcription factor containing a DNA binding domain called DM-domain [4]. In addition, pioneering studies using model organisms have demonstrated that sex-determining genes differ among species upstream of the hierarchies [4,5]. In contrast, little is known about the mechanisms of environmental sex determination (ESD) because organisms with ESD systems are poor genetic models.

**Fig 1:**
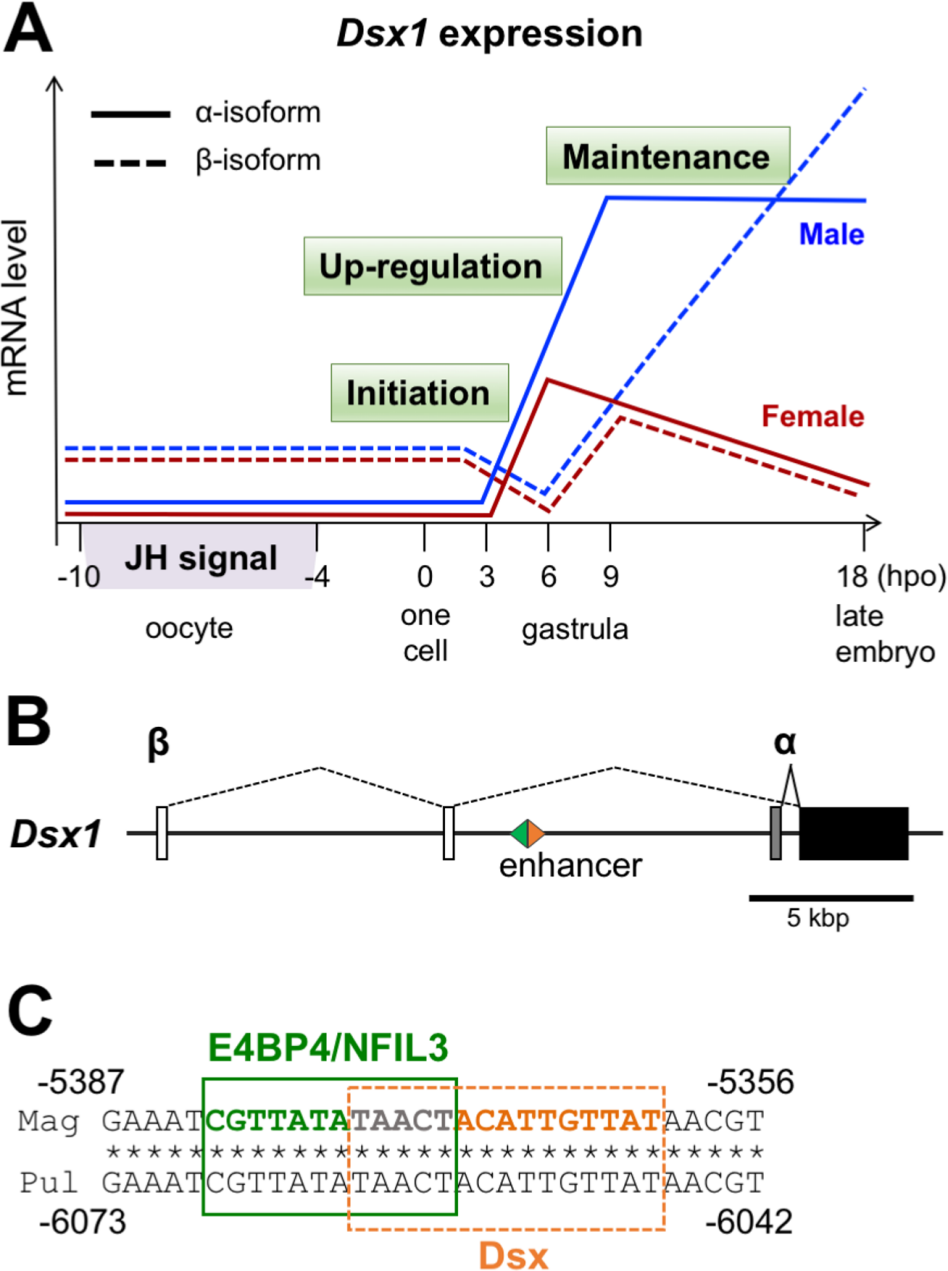
Regulation of *Dsx1-α* and *β* expression during embryogenesis. (A) Schematic representation of the expression patterns of *Dsx1-α* (solid line) and *β* (dash line) mRNAs at various developmental stages of female (red) and male (blue). Four to ten hours before ovulation is the critical period when the developing oocyte is sensitive to JH or JHA for commitment to male trait development. At 0 hpo (hour post oviposition), the embryo is at a one cell stage and only the maternal mRNA of β-isoform is detected both in female and in male. Before embryos become gastrula (initiation phase), non-sex specific *Dsx1-e* transcription starts and maternal *Dsx1-at*transcript is degraded. During gastrulation (up-regulation phase), *Dsx1-r* mRNA increases only in males, and later *Dsx1-in*expression also becomes male-specific. After gastrulation stage (maintenance phase), both isoforms show male-specific expression. (B) Position of the enhancer sequence on *D*. *magna Dsx1* locus. Since male-specific expression of *Dsx1-α* mRNA occurs earlier than *β*-isoform, the potential TFs binding sites were searched at the upstream region of *Dsx1-α* transcription start site. (C) Sequence of the enhancer that contains a bZIP protein binding (green box) site overlapped with a Dsx binding site (orange box). This sequence is conserved between *D*. *magna* (Mag) and *D*. *pulex* (Pul).

The crustacean waterflea, *Daphnia magna*, has emerged as a model organism for understanding ESD because of its fully sequenced genome [6,7] and advances in genetic manipulations through RNAi [8], ectopic expression [9], CRISPR/Cas9 [10] and TALEN systems [11–13]. In healthy populations, *Daphnia* normally produces female clones through parthenogenesis, but switches to sexual reproduction when environmental qualities for growth and reproduction decline [14]. In unfavorable environments such as shortened photoperiod, lack of food and/or high population density, *Daphnia* produces clonal males that allow fertilization of haploid eggs, which results in the production of resting eggs as a survival strategy upon harsh conditions [15]. We and others have shown that juvenile hormone analogs (JHAs) induce male production in cladoceran crustaceans without environmental cues [16,17]. A developing oocyte is sensitive to JH or JHA and a period when eggs are destined to be males by these chemicals is four to ten hours before ovulation (Fig 1A) [16,17], suggesting that environmental cues for sex determination are converted to JH signals neuroendocrinically. We also found that, during embryogenesis, a DM domain gene name *Doublesex1* (*Dsx1*) is exclusively expressed in male-specific tissues and regulates the male trait development in *D*. *magna* [18], which provides evidence that both GSD and ESD have the same origin and share similar genetic components in their sex-determining pathways.

To understand mechanisms of JH-dependent *Dsx1* activation in *D*. *magna*, we had previously examined temporal change of its expression during embryogenesis [18]. Of the two *Dsx1* mRNAs (*Dsx1*-α, *Dsx1-β*) which differ only at the 5′ UTR, zygotic transcription of *Dsx1-α* mRNA is largely divided into three phases (Fig 1A), non-sex specific transcription prior to early gastrula at 6-hour post ovulation (hpo) (initiation), male-specific activation during gastrulation from 6- to 9-hpo (up-regulation), and constant transcription during late embryogenesis (maintenance). Male-specific transcription of *Dsx1-pe*mRNA starts three hours later than *Dsx1-s* mRNA (around 9-hpo) and thereafter become more abundant in male embryos. We also generated transgenic *D*. *magna* to visualize spatiotemporal expression patterns and discovered that male-specific *Dsx1* expression starts in a presumptive primary organizer that migrates from the rostral to the caudal side on a ventral region at 11-hpo and thereafter gradually becomes specialized in male traits [19]. These previous findings suggest that JH activates *Dsx1-α* mRNA transcript in a specific population of gastrula cells. However, there is a significant time lag between the critical period of the JH action and onset of up-regulation of *Dsx1-α* mRNA levels (Fig 1), suggesting that *Dsx1* is not a primary JH-responsive gene regulated by the JH receptor protein, Methoprene-tolerant (MET) [20], but unknown transcription factors control its male-specific up-regulation in gastrula.

In this study, we aimed to identify the transcription factor responsible for male-specific up-regulation of *Dsx1-α* mRNA transcription that starts at 6-hpo. We searched for potential transcription factor binding sites at a region upstream of the transcription start site of *Dsx1-α* transcript. We found a potential enhancer that contains a consensus sequence of the Dsx binding site and an overlapping element for binding of an ortholog of the bZIP transcription factors, *Drosophila* Vrille (Vri) and vertebrate E4BP4/NFIL3, which are known to be involved in various general development processes including growth [21,22], circadian clock regulation [23,24], metamorphosis [25], apoptosis [26], and human T cell function [27]. In *D*. *magna*, *Vri* showed male-specific transient expression at 6-hpo. Loss- and gain-of-function analyses showed Vri to be necessary and sufficient for *Dsx1* activation. In addition, the disruption of the enhancer suggested Vri-dependent *Dsx1* activation. We infer co-option of the transcription factor *Vri* to the environmental sex-determination cascade.

## Results

### The E4BP4/NFIL3 ortholog *Vrille* is transiently activated by JH prior to *Dsx1* activation

To find candidate transcription factors (TFs) that activate *Dsx1* male-specific expression from 6-hpo, we analyzed a sequence within 7,899 base pairs upstream from the transcription start site of *Dsx1-α* mRNA. First, elements similar to known TF binding sites were searched with the TFBIND program [28] using the transcription factor database TRANSFAC R.3.4. Next, because *Dsx1* up-regulation and maintenance phases suggest positive feedback regulation of this gene, we investigated consensus binding sites of *Drosophila melanogaster* Dsx. Of the thousands of potential TF binding sites found in this study, we focused on an element similar to the fat body enhancer of the *Drosophila* yolk protein gene 1 that contains a Dsx binding site and an overlapping bZIP protein binding site [29]. We confirmed conservation of its position and sequence in the related daphniid species *Daphnia pulex* (Fig 1B and 1C). In this *Daphnia* species, a binding site for bZIP protein in the potential *Dsx1* enhancer matched to a consensus binding site for mammalian E4BP4/NFIL3, suggesting that an ortholog of E4BP4/NFIL3 may function as a transcriptional activator of *Dsx1* in *Daphnia*. To investigate the existence of an E4BP4/NFIL3 ortholog in *D*. *magna*, we performed a BLAST search using an amino acid sequence of the human E4BP4/NFIL3 against the *D*. *magna* genome database and found one ortholog that shows high homology in the bZIP domain to E4BP4/NFIL3 proteins (S1 Fig). We determined the cDNA sequence by 5′ and 3′ RACE reactions and obtained a 2,394 bp nucleotide sequence that codes for 797 amino acids (S2 Fig). Phylogenetic analysis using bZIP domains from various animals revealed that the *Daphnia* E4BP4/NFIL3 ortholog is most closely related to the insect E4BP4/NFIL3 ortholog Vrille (S3 Fig). Therefore, we designated this gene as *Vrille* (*Vri*).

**Fig 2:**
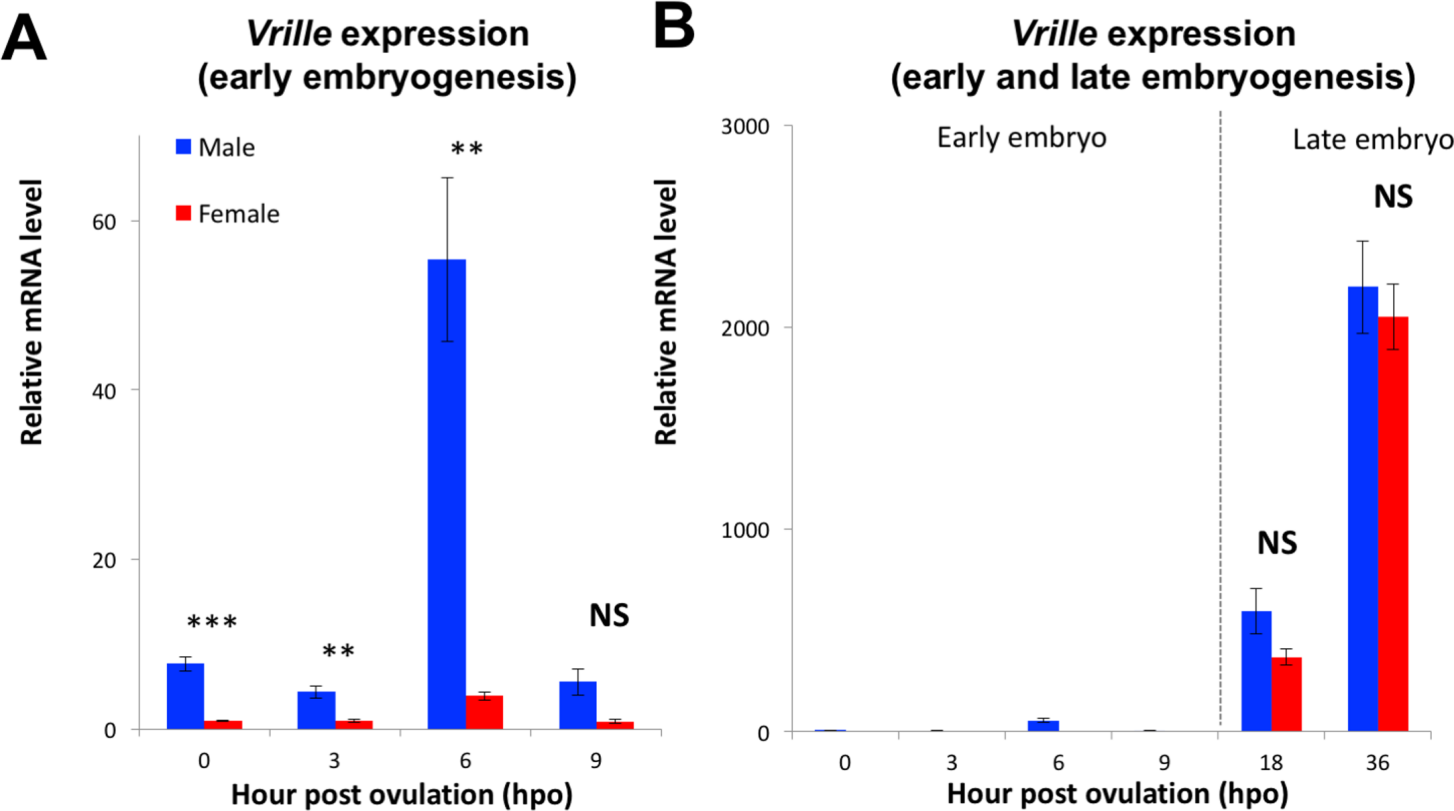
*Vrille* expression during embryogenesis of *D*. *magna*. (A) Sexual dimorphism of *Vri* expression at early embryonic stages (0, 3, 6, and 9-hpo). (B) Non-sex-specific *Vri* expression in the late embryos (18 and 36-hpo). The *Vri* expression levels were normalized to the reference gene expression levels (ribosomal protein L32). The normalized *Vri* expression in female embryos at 0-hpo was set to one. (Student's t-test; *, P<0.05; **, P<0.01; ***, P<0.001; NS = No significant different).

We then analyzed the temporal expression profile of *Vri* by qRT-PCR during embryogenesis (Fig 2). At early stages of embryogenesis (0, 3, and 6-hpo), *Vri* expression in males was higher than that in females. At 6-hpo, *Vri* transcripts transiently became more abundant and retained the sexually dimorphic expression pattern (Fig 2A). At later embryonic stages (18 and 36-hpo), *Vri* expression increased both in males and in females and lost its sexual dimorphism (Fig 2B).

### Vri functions as a transcription activator for *Dsx1* expression

The existence of a Vri binding site in the *Dsx1* promoter sequence and the male-specific expression of *Vri* prior to *Dsx1* up-regulation led us to hypothesize that *Vri* could regulate male-specific *Dsx1* up-regulation in gastrula. To investigate this hypothesis, we performed RNAi-mediated knockdown analysis as described previously [8,30]. To confirm specificity of phenotypes induced by *Vri* RNAi, we designed two siRNAs, Vri_siRNA_1 and Vri_siRNA_2, which differ at their target sequences (S2 Fig). To observe the cells and tissues influenced by *Vri* RNAi during embryogenesis, we used transgenic H2B-GFP expressing *Daphnia* that allows us to visualize individual cells in an embryo [31]. We injected each siRNAs into the eggs induced to become males by exposure to the JH agonist Fenoxycarb.

**Fig 3:**
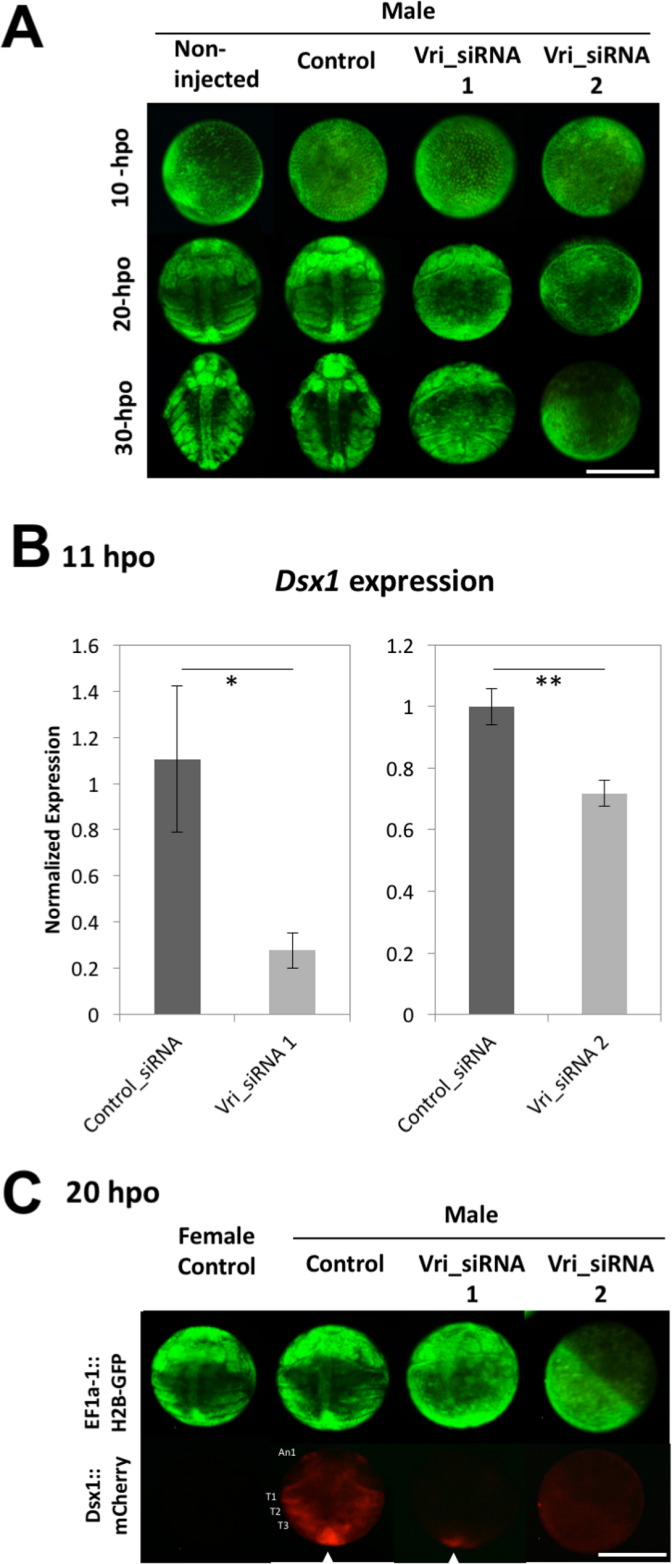
Effects of *Vri* RNAi on embryonic development and *Dsx1* expression. (A) Images of male embryos at 10, 20 and 30 hours after siRNA injection. Two *Vri* siRNAs (Vri_siRNA_1 and Vri_siRNA_2) were injected into transgenic lines and control siRNA was used as a negative control. The injected embryos were brighter compared to the non-injected ones because of the fluorescence from Lucifer yellow dye co-injected with siRNAs. (B) Spatial expression pattern of *mCherry* as a reporter of *Dsx1* gene expression at 20-hpo. An1, first antennae; T1, thoracic appendage 1; T2, thoracic appendage 2; T3, thoracic appendage 3; An arrowhead indicates the posterior growth zone; Scale bars: 200 μm. (C) The *Dsx1* expression level at 11 h after siRNA injection was measured by qRT-PCR. Control, Vri_siRNA_1 and Vri_siRNA_2 indicate control-, Vri_siRNA_1 and Vri_siRNA_2 injected males, respectively. Each experiment was performed in biological triplicate. For each replicate, nine or ten embryos were pooled in one tube, subjected to total RNA extraction and used for quantitation. (Student's t-test; *, P<0.05; **, P<0.01).

Based on H2B-GFP expression patterns, development of both Vri_siRNA_1- and Vri_siRNA_2-injected embryos seemed to be normal at around 10 to 11-hpo. At 20-hpo, Vri_siRNA_1-injected embryos developed cephalic appendages such as second antennae but did not start thoracic segmentation in contrast to control embryos (Fig 3A). Vri_siRNA_2-injected embryos died because of more severe phenotypes in which the segmental structures were not formed. At 30-hpo, Vri_siRNA_1-injected embryos showed abnormal segmentation of thoracic appendages, and undeveloped posterior and anterior regions of the embryos (Fig 3A), which prevented us from investigating sex-reversal in sexually dimorphic structures such as the 1st antennae. These RNAi-dependent severe deformities were also observed in females (S5 Fig).

To exclude the possibility that the developmental defect affects *Dsx1* expression, we analyzed *Dsx1* expression levels in RNAi embryos at 11-hpo by qRT-PCR and validated that *Vri* expression level was negligible in both of the RNAi embryos (S4 Fig). qRT-PCR analysis also revealed that both Vri_siRNA_1 and Vri_siRNA_2 reduced *Dsx1* expression (Fig 3B). To further analyze where *Vri* RNAi reduced *Dsx1* expression, we used another transgenic *Daphnia*, a *Dsx1* reporter strain that expresses mCherry, the red fluorescence protein under the endogenous *Dsx1* promoter/enhancer [19]. At 20-hpo, in control male and female *Daphnia*, the mCherry fluorescence appeared exclusively in male embryos and is localized in the 1st antennae, which are the first organs to show a male-specific trait in *Daphnia*. In addition, mCherry-expressing cells could be seen in thoracic appendages, which may be supplied from the posterior growth zone [19] (Fig 3C). In Vri_siRNA_1-injected male embryos, mCherry signal could be seen only in the posterior growth zone but its signal was weaker. Vri_siRNA_2 injected embryos did not show any red fluorescence (Fig 3C, Table 1), although due to severe effect of *Vri* silencing on embryonic processes, we could not exclude the possibility that some of the structures which normally express the mCherry reporter were not properly formed when *Vri* was silenced.

**Table 1:** Summary of *Vri* RNAi experiments using the *Dsx1* reporter strain.

siRNA	Sex	Injected	Hatched	Juvenile	Decline of mCherry fluorescence
Vri_siRNA_1	Male	20	0	0	90% (18/20)
Vri_siRNA_2	Male	9	0	0	88.9% (8 /9)
Control_siRNA	Male	8	8	8	0% (0/8)

To test whether transient expression of *Vri* in early embryos is sufficient to activate *Dsx1* and trigger male development, we induced transient ectopic expression of *Vri* in females by delivering capped, polyadenylated mRNAs into ovulated eggs via microinjection. We first attempted to establish a system to mimic transient expression of *Vri* in early male embryos. We constructed *GFP* mRNAs harboring the 5′ UTR and 3′ UTR sequences obtained from *Xenopus laevis β-globin* gene and injected this chimeric *GFP* mRNAs into female eggs. This injection led to expression at early embryogenesis (3 to 10-hpo) but not in the later stages (S6 Fig). Therefore, we linked the *X*. *laevis β-globin* UTRs to the *Vri* CDS and injected this chimeric *Vri* mRNA into wild-type eggs that would develop into females.

**Fig 4:**
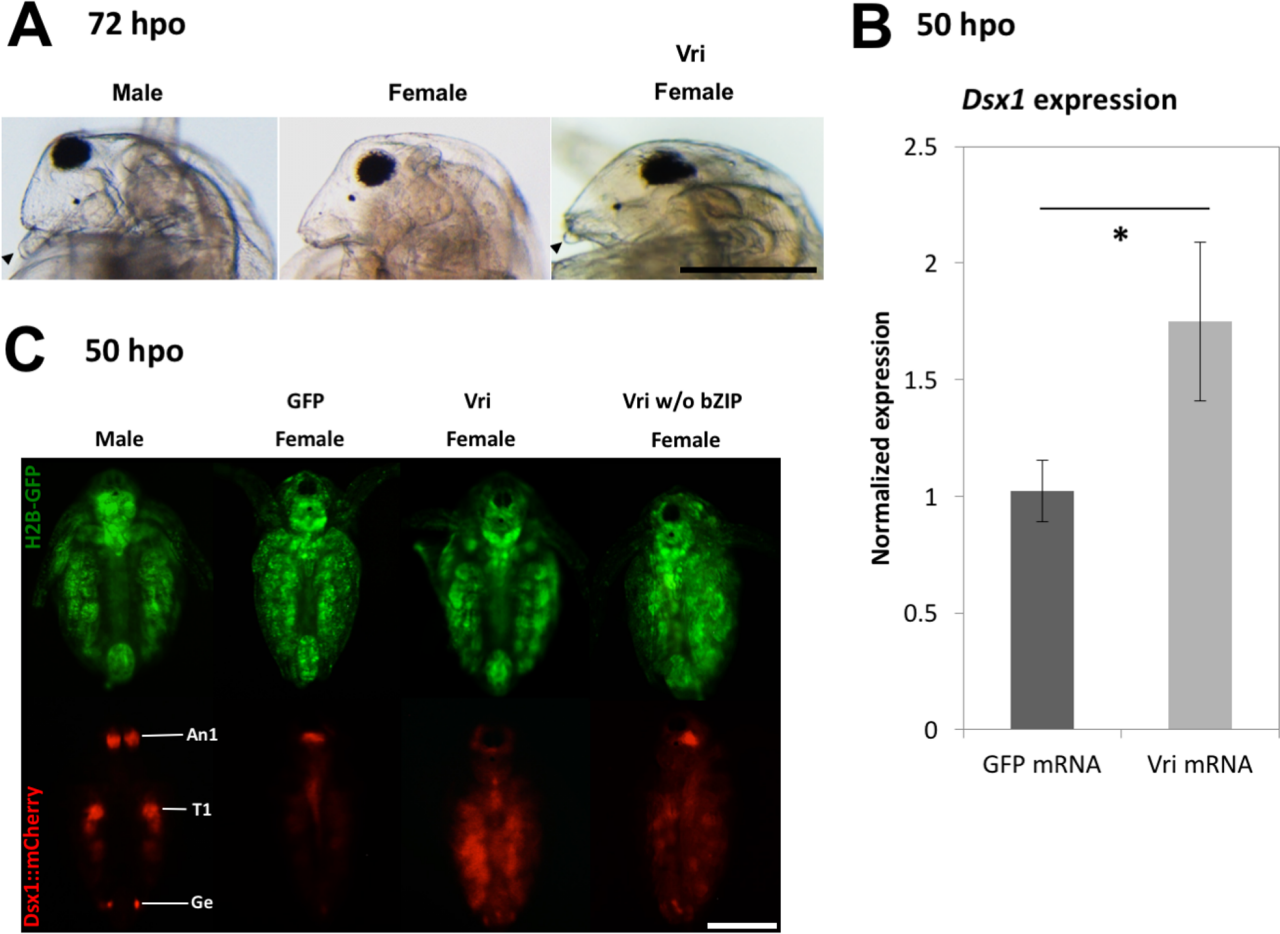
Masculinization of female embryos by *Vri* over-expression. (A) Phenotype of *Vri*-mRNA injected juveniles at 72-hpo. Black arrowheads indicate first antennae. (B) *Dsx1* expression level in *Vri* mRNA-injected embryos at 50-hpo. This experiment was performed with four biological replicates. For each replicate, one daphniid was subjected to total RNA extraction and used for quantitation. (Student's t-test; *, P<0.05). (C) Spatial expression pattern of *mCherry* as a reporter of *Dsx1* gene expression at 50-hpo. Non-injected males display mCherry expression in the first antenna (An1), first thoracic leg (T1) and genital (Ge). GFP-, Vri- and Vri without bZIP indicate negative control female injected with *GFP* mRNA, females injected with *Vri* mRNA harboring its full-length CDS, and females injected with mRNA encoding bZIP domain-lacking Vri, respectively. Scale bars: 200 μm.

Although this chimeric mRNA induced high embryonic lethality (Table 2), the juveniles that survived showed partial elongation of the 1st antennae in an mRNA concentration-dependent manner (Fig 4A, Table 2). Consistent with this masculinized phenotype, we could confirm up-regulation of *Dsx1* expression levels in *Vri* RNA-injected daphniids by qRT-PCR at 48 to 50-hpo (Fig 4B). Low viability prevented us from evaluating further masculinization in injected female animals.

**Table 2:** Masculinization of 1^st^ antennae by *Vri* overexpression in females.

mRNA	Conc. (ng/μL)	Injected eggs	Swimming juveniles	Viability	Elongated 1^st^ antennae
***Vrille***	1000	64	8	13%	75% (6/8)
	250	34	6	18%	67% (4/6)
	62.5	16	5	31%	0% (0/5)
***GFP***	800	29	24	83%	0% (0/24)

In addition, by using the *Dsx1* reporter strain, we tested the effects of the same chimeric *Vri* mRNA on *Dsx1* activation in females and detected high and widespread *mCherry* expression mainly in thoracic appendages at 50-hpo (Fig 4C, Table 3). To confirm whether Vri’s DNA binding activity was necessary for *Dsx1* activation, we injected mRNA encoding a mutated form of Vri that lacked the bZIP domain (S2 Fig). This mutated Vri could increase *Dsx1* expression levels but showed lower transactivation activity (Fig 4C, Table 3). Taken together, these loss-and-gain-of-function analyses show that Vri functions as a transcription activator for *Dsx1* expression in *D*. *magna*.

**Table 3:** Activation of *mCherry* by *Vri* overexpression in female embryos from the *Dsx1* reporter strain.

mRNA	Concentration	Red fluorescence at 20-hpo
*Vrille* *(full length)*	1 μg/μl	100% (5/5)
	250 ng/μl	100% (12/12)
	62.5 ng/μl	100% (13/13)
*Vrille* *(w/o bZIP)*	1 μg/μl	80% (8/10)
*GFP*	1 μg/μl	0% (0/9)

### Disruption of the enhancer reduced *Dsx1* expression in males

**Fig 5:**
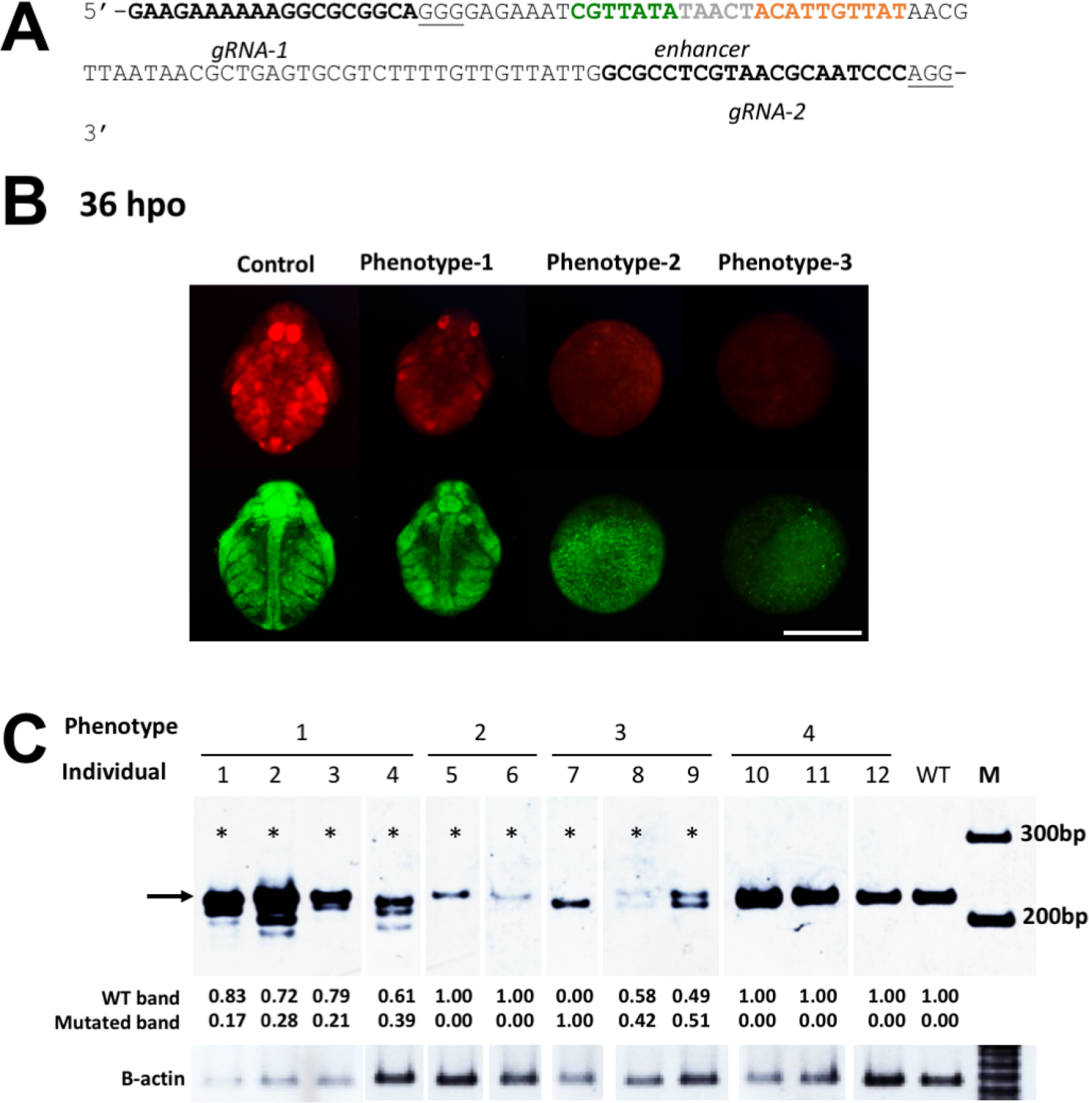
Disruption of the enhancer region by the CRISPR/Cas9 system. (A) The sequences of gRNA target sites (bold) located near the enhancer region. The underlines show PAM sequences. (B) Spatial expression pattern of *mCherry* as a reporter of *Dsx1* gene expression at 36-hpo. The Cas9 protein was co-injected with a pair of gRNA-1 and gRNA-2 into 12 male embryos of the *Dsx1* reporter strain. Scale bar: 200 μm (C) PAGE analysis of PCR products by genomic PCR to amplify a region including the enhancer. The original size of the PCR products should be 214 bp, indicated by the black arrow. Asterisks show the individual that contains mutations. The numbers under the image of the PAGE gel indicate intensity of wild-type (WT) and mutated band measure by ImageJ application. M denotes DNA marker.

To test whether the enhancer element is required for *Dsx1* activation and male trait development, we tried to disrupt its sequence on the genome by using the CRISPR/Cas9 system. Because the low GC content (23%) of the enhancer prevented us from designing enhancer-targeting TALENs and gRNAs, we designed two separate gRNAs, gRNA-1 and gRNA-2, near to the enhancer (Fig 5A) and confirmed the functionality of the gRNAs by Cas9 *in-vitro* cleavage assay (S7 Fig). We then co-injected the two gRNAs with Cas9 protein into the *Dsx1* reporter strain [19] that would develop into males and evaluated effects of enhancer disruption on *Dsx1* and the morphological phenotypes. At 36-hpo, we found four different phenotypes from the 12 injected embryos. Four embryos (#1, #2, #3, and #4) exhibited phenotype-1 (Fig 5B), in which embryonic development was delayed and the embryos showed weaker mCherry fluorescence than control but at later stages, they could have normal male traits development. Two embryos (#5 and #6) showed phenotype-2 wherein egg development was disturbed and mCherry signal was weak with abnormal localization. Phenotype-3 was observed in three embryos (#7, #8, and #9) showing the most severe deformities and no mCherry expression. The remaining three embryos (#10, #11, and #12) showed no apparent change in phenotype compared to control (Phenotype-4). The abnormal development of these eggs prevents us from observing the sex-specific traits or feminized phenotypes.

To examine the correlation between the introduced mutations and the observed phenotypes, we extracted genomic DNA from each embryo and performed genomic PCR to amplify the enhancer region. Native PAGE electrophoresis of PCR products showed either bands of smaller sizes than what was expected from wild-type sequence, or wild-type bands of reduced intensity, suggesting that large and small deletions in the enhancer region had occurred (Fig 5C). We measured the intensity of each band and calculated the ratio of intensity of expected to smaller bands, and observed that the more severe the phenotype of injected embryo was, the higher was the ratio. These results indicate that the enhancer may be a *cis*-regulatory element for male-specific *Dsx1* expression.

In addition, we attempted to generate the enhancer knockout mutants by injecting the Cas9 protein-gRNAs complexes and collecting offspring of the injected daphniids. In injection into eggs that develop into females, neither somatic nor heritable mutations were detected (S1 Table, S2 Table). In male daphniids, the injection led to high embryonic lethality (>90%) (S2 Table). We could collect offspring by feminizing the survived males using *Dsx1* RNAi, but no mutant line was generated.

## Discussion

We had previously found that JH and *Dsx1* are essential for environmental sex determination in *D*. *magna* [18]. JH drives commitment to male development in oocytes at 4 to 10 h before ovulation [32]. In response to JH signal, *Dsx1* is up-regulated from early gastrula at 6 h post-ovulation and is maintained in late embryos for the control of male trait development [19]. However, the molecular mechanisms that mediate JH signaling and *Dsx1* up-regulation have remained unknown. In this study, we identified the bZIP transcription factor, Vri as a candidate transcriptional activator by sequence analysis of the *Dsx1* promoter/enhancer. Further studies involving expression pattern analysis, loss- and gain-of-function analyses and disruption of an enhancer harboring a Vri consensus binding site indicated that it is required for male-specific *Dsx1* up-regulation. Our findings provided evidence that Vri has been co-opted as a component upstream of *Dsx1* in the environmental sex-determining pathway.

Over the past several years, new sex-determining genes have been identified in genetic sex-determining pathways in several animals, which reveals the importance of gene co-option. Mechanisms for co-option of new sex-determining genes are largely divided into three categories: 1) allelic diversification, 2) duplication of genes related to sexual development and 3) recruitment of a novel gene with no homology to any known sexual regulators [33]. First, by allelic diversification, transcription factor SOX3 was recruited as a master regulator for sex determination in mice [34] and Indian ricefish [35]. By the same mechanism, the DM-domain gene Dmrt1 and the gonadal soma-derived growth factor (Gsdf) were also co-opted at the top of sex-determining pathways in birds [36] and Luzon ricefish [37] respectively. Second, in frog [38] and Medaka [39], the *Dmrt1* gene was duplicated and one of the duplicates gained function as a master sex-determining gene. In insects, transformer orthologs that are conserved components of the sex-determining cascades, were duplicated in honeybee [5,40], resulting in upstream regulators named the *Csd*. These findings suggested that orthologous genes are repeatedly co-opted for genetic sex-determining pathways in independent animal lineages [33] even though, in the silkworm and the rainbow trout, novel factors, a piRNA [41] and the interferon regulatory factor irf9 [42] seems to have evolved as sex determiners. The *Vri* gene was previously identified as one of genes regulated by Dsx in male *Drosophila* [43]. As well as most of previously identified sex-determining genes, *Vri* might be repeatedly employed in the sex-determining regulatory networks. In environmental sex-determining *D*. *magna*, without allelic diversification and duplication, *Vri* would have been co-opted in upstream of Dsx1. Sex-related roles of *Vri* in various organisms should be examined in future.

Our findings indicate that Vri functions as an activator of the *Dsx1* gene in *Daphnia*. In *Drosophila*, Vri regulates various developmental processes such as cell growth, proliferation and flight [21,44], as well as metamorphosis [25] and tracheal integrity [22]. In addition to these processes, Vri is required for circadian oscillation by repression of *Clock* transcription [24]. In mammals, the Vri ortholog E4BP4/NFIL3 is also reported as a clock-controlled gene. It competes for the binding site of the PAR-protein. Both *Drosophila* Vri and mammalian E4BP4/NFIL3 are well known as transcriptional repressors. However, in the human immune response system, E4BP4/NFIL3 was identified as an activator of the *IL3* promoter [27] and was also shown to up-regulate *IL-10* and *IL-13* [45]. It is essential for lineage commitment of innate lymphoid cells (ILCs) [46]. In natural killer cell development, E4BP4/NFIL3 interacts with the histone ubiquitinase MYSM1 and maintains an active chromatin state at the *Id2* locus [47]. In *Daphnia* sex determination, Vri works at the gastrulation stage when lineage commitment occurs. These similarities in regulation at the genetic and cellular levels may suggest that the molecular mechanism of Vri-dependent *Dsx1* activation is similar to that of E4BP4/NFIL-3 function in human ILCs.

**Fig 6:**
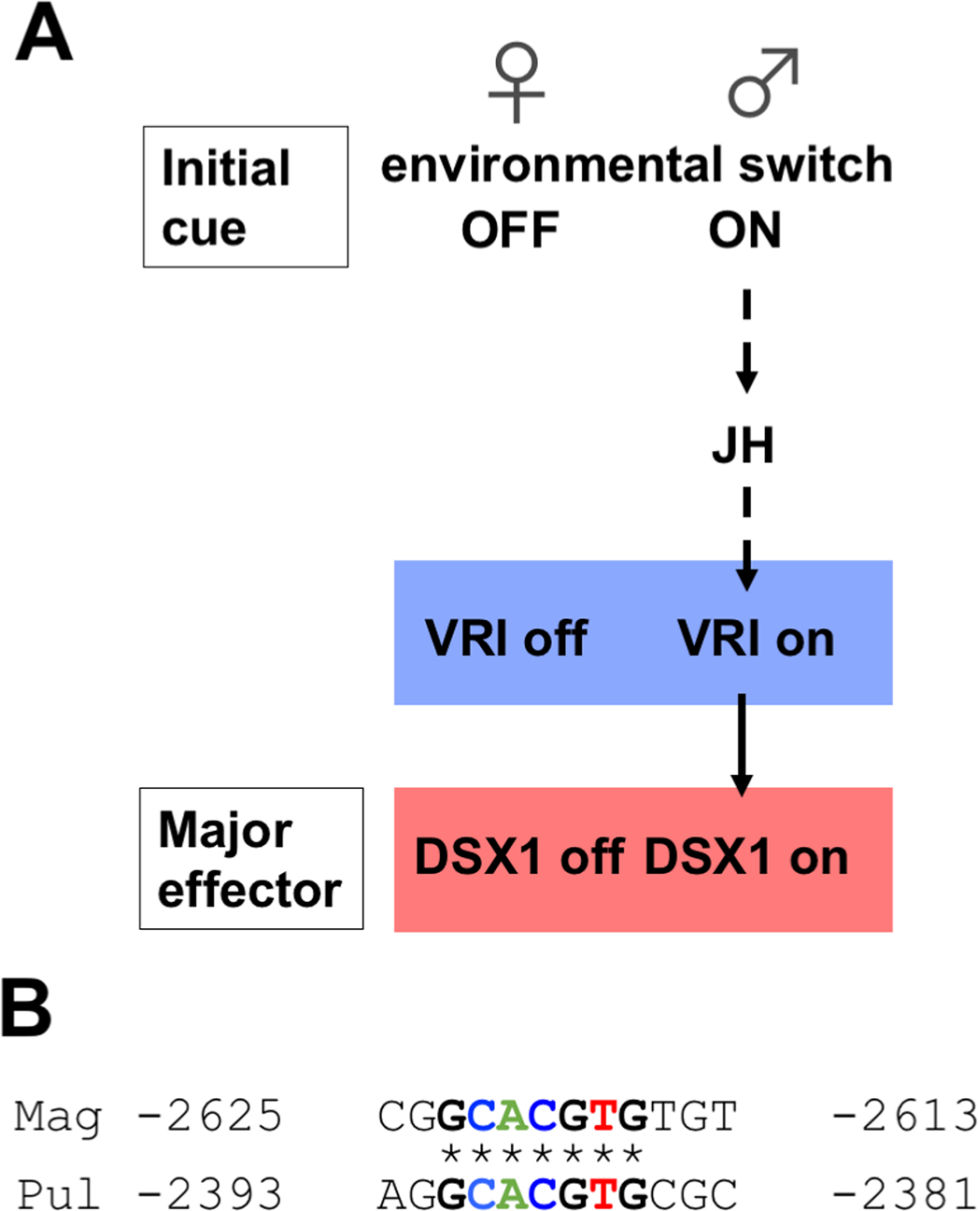
Position of Vri in *Daphnia* environmental sex-determining pathway. (A) Simplified view of the sex-determining pathway in *D*. *magna*. Solid arrow indicates direct interaction whereas dash arrows indicate possible interactions. (B) JHRE motif in *Daphnia Vri* promoter/enhancer. To identify the motif, we used the JHRE consensus sequence mentioned in [57]. The JHRE motif was conserved in *D*. *magna* (Mag) and *D*. *pulex* (Pul).

Based on the timing of action of JH, Vri, and Dsx1, we were able to propose a hierarchy of signal transduction in environmental sex determination (Fig 6A). In this hierarchy, JH first stimulates expression of *Vri*, which in turn activates *Dsx1* expression. To examine the possibility that the JH-receptor MET directly regulates *Vri* activation, we searched for sequences similar to the MET-binding site for the *Vri* promoter/enhancer and found one candidate sequence that is conserved in two *Daphnia* species (Fig 6B), suggesting that this motif functions as an element to regulate the JH-dependent gene expression. However, because there is still time lag between JH signaling and *Vri* activation, there might be other molecules that respond to JH signal and then direct the male-specific *Vri* transcription. Thus, discovering these early response genes of JH signal may improve our understanding of hormonal signaling and the environmental sex determination pathway.

Interestingly, in the initiation phase of *Dsx1* transcription, *Dsx1* is transcribed both in males and in females at 3 to 6-hpo. We hypothesize that in males, Vri might form a heterodimer with Dsx1, bind to the enhancer and up-regulates *Dsx1* expression at 6 to 9-hpo. *Drosophila* Dsx is known to form a heterodimer with the bZIP-domain transcription factor and binds to the fat body enhancer (FBE) of the yolk protein gene [29]. Transactivation of *Dsx1* by a truncated Vri lacking the bZIP domain in this study also suggests that heterodimer formation allowed the mutated Vri to access the target binding site. These suggest that the heterodimeric combination of Dsx and bZIP transcription factors has functioned as a transcriptional regulator before divergence of insects and crustaceans. Even though we provide substantial genetic evidence of *Dsx1* activation by Vri in early embryos, because the loss- and gain-of- *Vri* function led to embryonic lethality, we still cannot conclude that Vri is the sole upstream component acting as a *Dsx1* activator that is necessary for male trait development. To understand more about Vri function in environmental sex determination, we will need to clarify localization of Vri in early embryos and perform knockdown/overexpression in cells that express Vri endogenously, which would avoid alternation of non-sex specific functions of Vri in later embryos.

In targeted mutagenesis using Cas9, we could not introduce any mutation into the Vri binding site at the *Dsx1* promoter/enhancer on the genome in females. In contrast, this mutagenesis introduced deletion at the target site on the genome in males and reduced *Dsx1* expression. These results suggest that this enhancer may be silenced via closed chromatin in females but is required for *Dsx1* activation in males. We also found that deletion of the enhancer led to embryonic lethality in males although we could not shed light on the mechanism underlying this high mortality. However, these clear differences of phenotypes between males and females in our targeted mutagenesis experiments indicate a male-specific role of this enhancer. Further study is needed to understand the epigenetic regulation at *Dsx1* locus.

In conclusion, we demonstrate co-option of the bZIP transcription factor Vrille upstream of the *Dsx1* in the environmental sex-determining cascade of the crustacean *D*. *magna*. *Vri* is transiently expressed in early gastrula in response to juvenile hormone and controls male-specific up-regulation of *Dsx1* in late gastrula. This is the first finding that *Vri* is recruited into sex determining pathways. Our finding reveals the remarkably plastic nature of *Dsx* regulation, which will contribute to understanding of the diversity and evolution of the sex-determining pathways in organisms.

## Materials and methods

### *Daphnia* strains and transgenic lines

All of the wild-type (WT) and transgenic lines share the same genetic background (NIES clone). They were cultured in ADaM medium [48] as described previously [49]. Male *Daphnia* were obtained by exposing female adults (2–3 weeks old) to 1 μg/L of the synthetic juvenile hormone analog, Fenoxycarb (Wako Pure Chemical; Osaka, Japan) [50]. We utilized previously established transgenic lines of *D*. *magna*. One of the transgenic lines (HG-1) expresses H2B-GFP protein under the control of *D*. *magna Elongation Factor 1 α-1* (*EF1α-1*) promoter/enhancer [31]. Another was the *Dsx1* reporter strain, which was generated by introducing *mCherry* gene upstream of *Dsx1* coding sequence in the genome of the HG-1 [19].

### Quantitative RT-PCR

Total RNA was extracted from female and male embryos in triplicates using Sepasol-RNAI solution (Nacalai Tesque; Kyoto, Japan). The RNA was subjected to cDNA synthesis using random primers (Invitrogen; Carlsbad, CA, USA) and the SuperScriptIII Reverse Transcriptase (Invitrogen). qPCR was conducted with the SYBR GreenER qPCR Supermix Universal (Invitrogen) using the Mx3005P real time (RT)-PCR system (Agilent Technologies; Santa Clara, CA, USA). *Vri* expression was quantitated and was normalized with the ribosomal protein *L32* expression level using the primers listed in S3 Table. The primers used to amplify the *Dsx1* and the ribosomal protein *L32* gene were the same as described previously [18]. For normalization of *Dsx1* expression level in the *Vri* knockdown using Vri_siRNA_1 and overexpression, expressions of three other reference genes, ribosomal *L8* gene, *β-actin* gene and *Cyclophilin* gene [51] were analyzed using the primers listed in S3 Table. The geometric mean of the reference genes were calculated and used for normalization as described previously [52].

### Cloning and sequencing of *Vri* gene

The *Vri* cDNA sequence was amplified from *Daphnia* by 5′ and 3′ rapid amplification of cDNA ends (RACE) methods as described previously [30]. The primer sequences used for cDNA fragment amplification were as follows: Vri 5′ RACE gene specific primer (5′-TGTTGCTGCCGATTGCGCTGACACTG-3′); Vri 5′ RACE nested primer (5′-CTCGGTCGAACGCCGTCCGCTACTG-3′); Vri 3′ RACE gene specific primer (5′-CCGGCCGTGTACTGCCGCTCAAACTA-3′); and Vri 3′ RACE gene nested primer (GGCTGCCGCTGTTCTGCTGACACTCA-3′). The resulting PCR products were excised from an agarose gel after electrophoresis, purified and were cloned into a TOPO vector (Invitrogen) for sequencing analysis. We then used the DNA sequence for homology search and phylogenetic analyses using BLAST and MEGA (version 7.0.21) as mentioned previously [30]. The Vri cDNA sequence is available from the DDBJ database (http://getentry.ddbj.nig.ac.jp/getentry/na/LC230164/?format=flatfile&filetype=html&trace=true&show_suppressed=false&limit=10) (Accession number LC230164).

### RNA preparation and microinjection

To knockdown the *Vri* gene, 100 μM of Vri_siRNA_1 and Vri_siRNA_2 (sequences indicated in S2 Fig) were used. A previously used control siRNA (5′- GGUUAAGCCGCCUCACAUTT-3′) was utilized as a negative control [53]. The siRNA oligonucleotides were dissolved in DNase/RNase-free water (Life Technologies Inc.; Grand Island, NY, USA).

To overexpress the *Vri* gene, chimeric *Vri* cDNA harboring the 5′ UTR and 3′ UTR of *X*. *laevis β-globin* gene was designed and subcloned downstream to the T3 promoter on the pRN3 vector [54]. The *Vri* CDS of this plasmid was replaced with the CDS of *GFP* fused with minos transposase for preparation of control mRNA for investigating effects of *β-globin* UTRs on mRNA stability and/or translation efficiency. These plasmids were linearized by BsaAI restriction enzyme, purified with phenol/chloroform extraction and used as templates for mRNA synthesis. *In vitro* transcription by T3 RNA polymerase and poly-A tail addition were performed according to the manufacturers’ protocol of the commercial kits mMessage mMachine T3 kit (Life Technologies Inc.) and Poly(A) Tailing kit (Life Technologies Inc.), respectively. The synthesized mRNAs were column purified by RNeasy Mini kit (Qiagen; Tokyo, Japan), followed by phenol/chloroform extraction, ethanol precipitation, and dissolution in DNase/RNase-free water.

For the syntheses of gRNAs, the templates were prepared by the cloning free method [55]. The sense synthetic oligo contains three main parts: a T7 promoter (shown in bold), a variable targeting sequence (N_18_) and the first 20 nt of the Cas9 binding scaffold sequence. The full sequence is as follows: (5′- GAAA**TTAATACGACTCACTATAGG**NNNNNNNNNNNNNNNNNNGTTTTAGAGCTAGAAATAGC-3′). The anti-sense oligo contains 80 nt full sequence of the Cas9 binding scaffold: (5′-AAAA GCACCGACTCGGTGCCACTTTTTCAAGTTGATAACGGACTAGCCTTATTTTAACTTGCTATTTCTAGCTCTAAAAC-3′) where the underlined nucleotides denote the complementary sequence between two oligo sequences. The PCR reaction was performed with PrimeSTAR polymerase (Takara Bio; Shiga, Japan). After purification by phenol/chloroform extraction, the DNA fragments were used as templates for in vitro transcription with the MEGAscript T7 kit (Life Technologies Inc.), followed by column purification with mini Quick Spin RNA columns (Roche diagnostics GmbH; Mannheim, Germany), phenol/chloroform extraction, ethanol precipitation, and dissolution in DNase/RNase-free water.

Microinjection was performed as described previously [8]. Eggs were obtained from adult *Daphnia* at 2–3 weeks of age, directly after ovulation and placed in ice-cold M4 media contained 80 mM sucrose. The specific RNAs for each experiment were mixed with either Alexa Fluor 568 dye (Life technologies Inc.) or Lucifer Yellow dye (Life technologies Inc.) with final concentrations of 0.01 μM and 1 μM respectively, as an injection marker. The microinjection was performed on ice and the injected eggs were incubated in a 96-well at 23°C for the appropriate time.

### Targeted mutagenesis and genotyping

We mixed *in vitro* synthesized RNA with Cas9 protein to make gRNA-Cas9 complexes. Cas9 protein was prepared as described previously [56]. They were incubated for 5 min at 37°C and injected into wild type *D*. *magna* eggs, as described previously [8]. To characterize the somatic mutation on Vri binding site generated by Cas9 protein, target loci were amplified by PCR from genomic DNA isolated from each injected egg. To extract the genomic DNA, injected embryos were homogenized individually in 90 μL of 50 mM NaOH with zirconia beads. The sample was heated at 95°C for 10 min, followed by a neutralization step by adding 10 μL of 1 M Tris-HCl (pH 7.5). Before this DNA extract was used as a PCR template, the sample was centrifuged at 13,000 g for 5 min. The PCR was performed with HS Ex Taq polymerase (Takara Bio) using a primer pair designed as follows: Vri-bs forward (5′-GATGTCACGAAATCTGAGGTC-3′) and Vri-bs reverse (5′-GATCTAAACACCTTGGCGTAAC-3′), which amplified 214 bp including the enhancer region. The PCR products were analyzed with native PAGE gel electrophoresis. To characterize the heritable mutagenesis, injected *Daphnia* were cultured separately until they produced offspring. The offspring were pooled (up to 8–10 daphniids) and genomic DNA extraction and genomic PCR were performed as mentioned above.

## Supporting information

S1 Fig Alignment of the bZIP domain of Vri and E4BP4/NFIL3 proteins. Dmag is *D*. *magna*, Tc is *T*. *castaneum* (beetle), Dp is *D*. *plexippus* (monarch butterfly), Dm is *D*. *melanogaster* (fruit fly), Dr is *D*. *rerio* (zebrafish), Mm is *M*. *musculus* (mouse) and Hs is *H*. *sapiens* (human).
(TIFF)

S2 Fig Nucleotide and deduced amino acid sequences of *D. magna Vri*. Black shaded amino acids indicate the putative bZIP domain and grey shaded nucleotide is the sequence that being removed for *Vri* over-expression experiment (*Vri* mRNA without bZIP domain). The underlined sequences are the target sites for Vri_siRNA_1 and Vri_siRNA_2 accordingly.
(TIFF)

S3 Fig Phylogenetic tree using the amino acid sequences of the bZIP domains of Vri and E4BP4/NFIL3 transcription factors. The percentages of the replicated tree in which the associated taxa clustered together in the bootstrap test (1,000 replicates) are shown next to the branches. The bar indicates branch length and corresponds to the mean number of the differences (P<0.05) per residue along each branch.
(TIFF)

S4 Fig Gene expression profile of *Vri* in embryos injected with control, Vri_siRNA_1 and Vri_siRNA_2 siRNAs at 11-hpo. (Student's t-test; **, P<0.01).
(TIFF)

S5 Fig Phenotypes of siRNA-injected female embryos at 10, 24 and 30 hours after injection. Scale bar: 200 μm.
(TIFF)

S6 Fig Temporal change of fluorescence in embryos injected with *GFP* mRNA harboring *X. laevis β-globin* UTRs. GFP was fused with Minos transposase, which resulted in nuclear localization of GFP as previously reported [54]. Scale bar: 200 μm.
(TIFF)

S7 Fig Gel electrophoresis after the Cas9 protein *in-vitro* cleavage assays. To examine the ability of designed gRNAs and Cas9 protein executing double strand break, 300 ng of plasmids harboring the target sequence were incubated with 1 μM Cas9 protein and 2 μM gRNA at 37°C for 1 hour in reaction buffer that contains 20 mM hepes (pH 7.5) 150 mM KCl, 0.5 mM DTT, 0.1 mM TCEP and 10 mM MgCl_2_. This reaction was stopped by adding 0.5 M EDTA to the reaction mixture. Cleavage of the plasmid by Cas9 and gRNA was observed by running the gel electrophoresis. The positive result can be interpreted by detecting the linear band of the plasmid on the gel. The target sequence for each gRNA (gRNA-1 and -2) is indicated in Fig 5. 2X means 2 μM Cas9 protein or 4 μM gRNA.
(TIFF)

S1 Table Somatic mutagenesis in female for disrupting the *Dsx1* enhancer by CRISPR/Cas9 system. (DOCX)

S2 Table Summary of heritable mutagenesis in female and male for disrupting the *Dsx1* enhancer. To evaluate heritable mutagenesis efficiency, we screened founder animals that produced progenies by genotyping. To feminize injected males, 100 μM *Dsx1* siRNA was co-injected with Cas9 protein and gRNAs.
(DOCX)

S3 Table Primer sequences for quantitative real-time PCR. (DOCX)
