# Supplementary figures and images for: Co-option of the bZIP transcription factor Vrille as the activator of Doublesex1 in environmental sex determination of the crustacean Daphnia magna
Source: PLoS Genet. 2017 Nov 2;13(11):e1006953. doi: 10.1371/journal.pgen.1006953 (PMC5667737; doi:10.1371/journal.pgen.1006953)

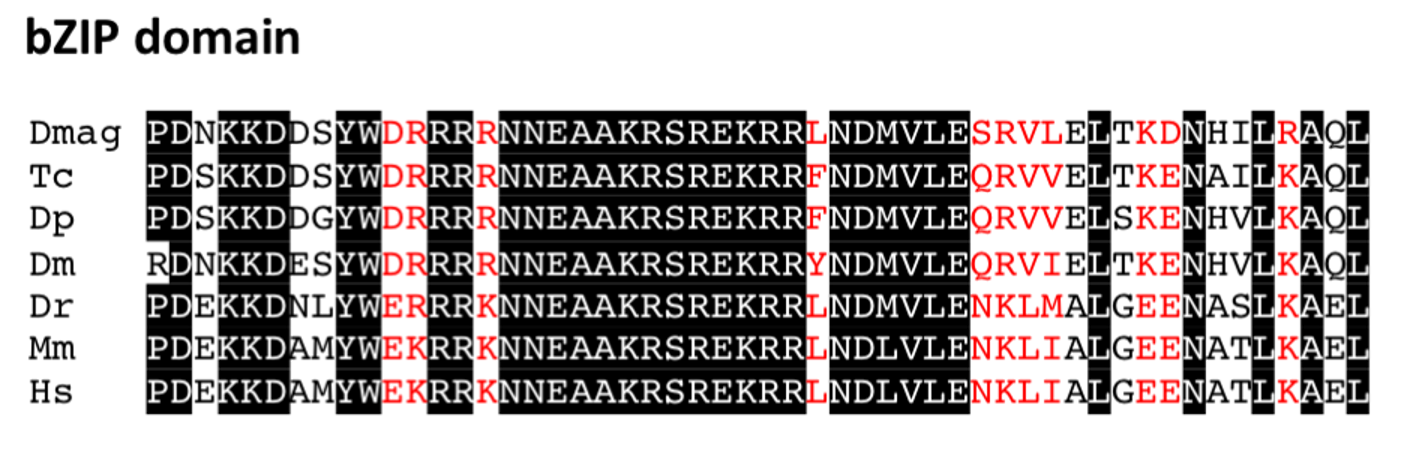

Supplement: S1 Fig — Dmag is D. magna, Tc is T. castaneum (beetle), Dp is D. plexippus (monarch butterfly), Dm is D. melanogaster (fruit fly), Dr is D. rerio (zebrafish), Mm is M. musculus (mouse) and Hs is H. sapiens (human). (TIFF) [file pgen.1006953.s001.tiff]

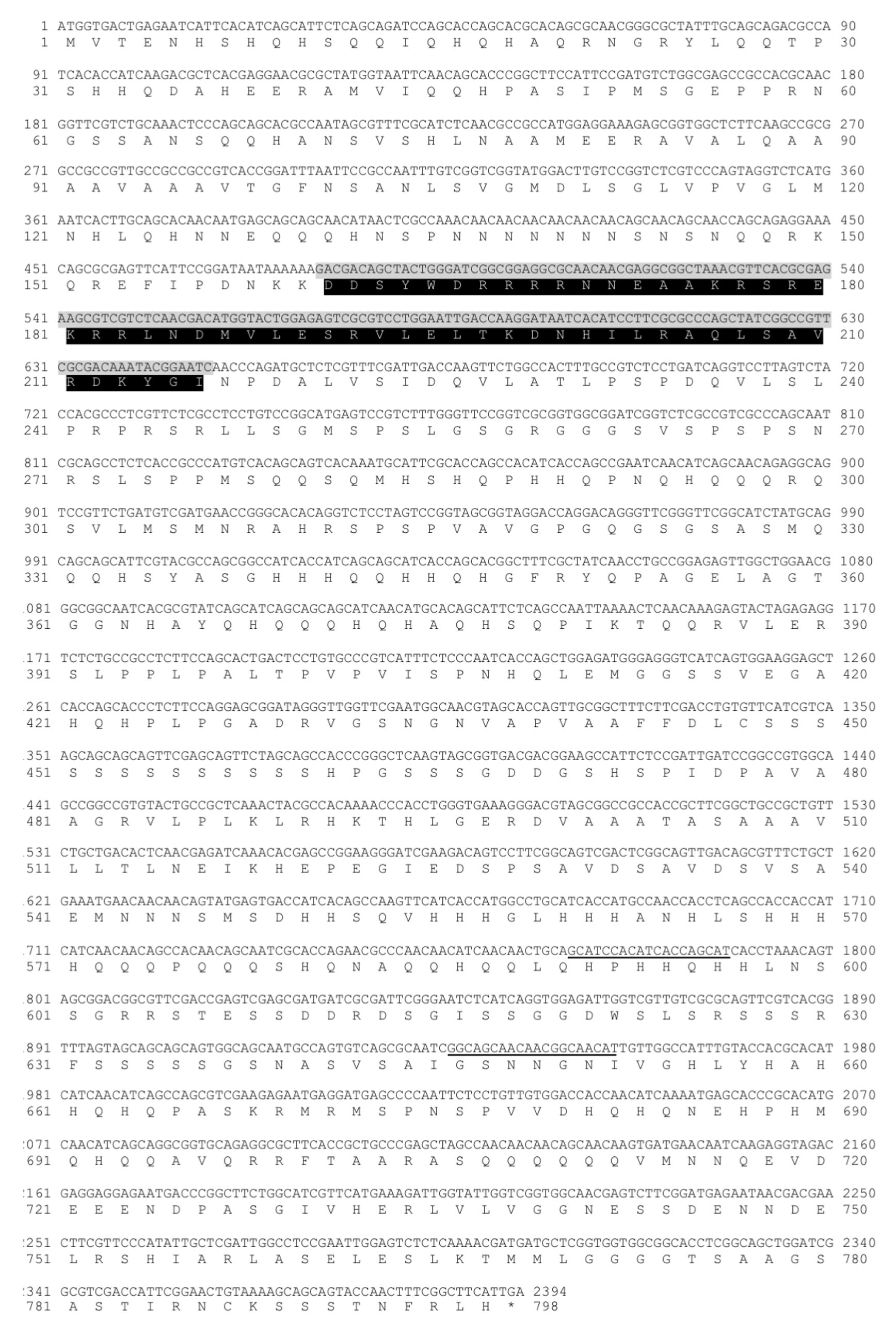

Supplement: S2 Fig — Black shaded amino acids indicate the putative bZIP domain and grey shaded nucleotide is the sequence that being removed for Vri over-expression experiment (Vri mRNA without bZIP domain). The underlined sequences are the target sites for Vri_siRNA_1 and Vri_siRNA_2 accordingly. (TIFF) [file pgen.1006953.s002.tiff]

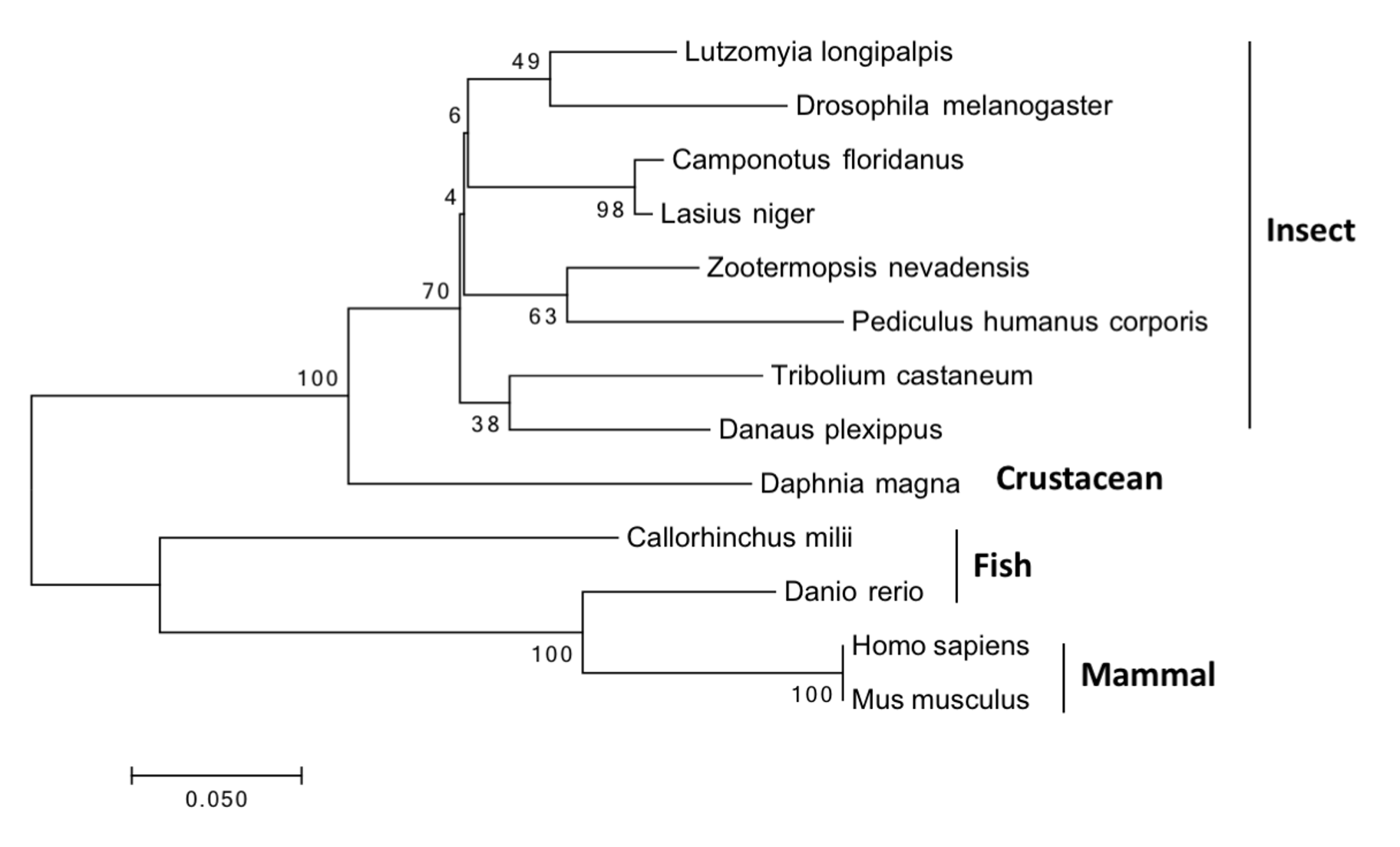

Supplement: S3 Fig — The percentages of the replicated tree in which the associated taxa clustered together in the bootstrap test (1,000 replicates) are shown next to the branches. The bar indicates branch length and corresponds to the mean number of the differences (P<0.05) per residue along each branch. (TIFF) [file pgen.1006953.s003.tiff]

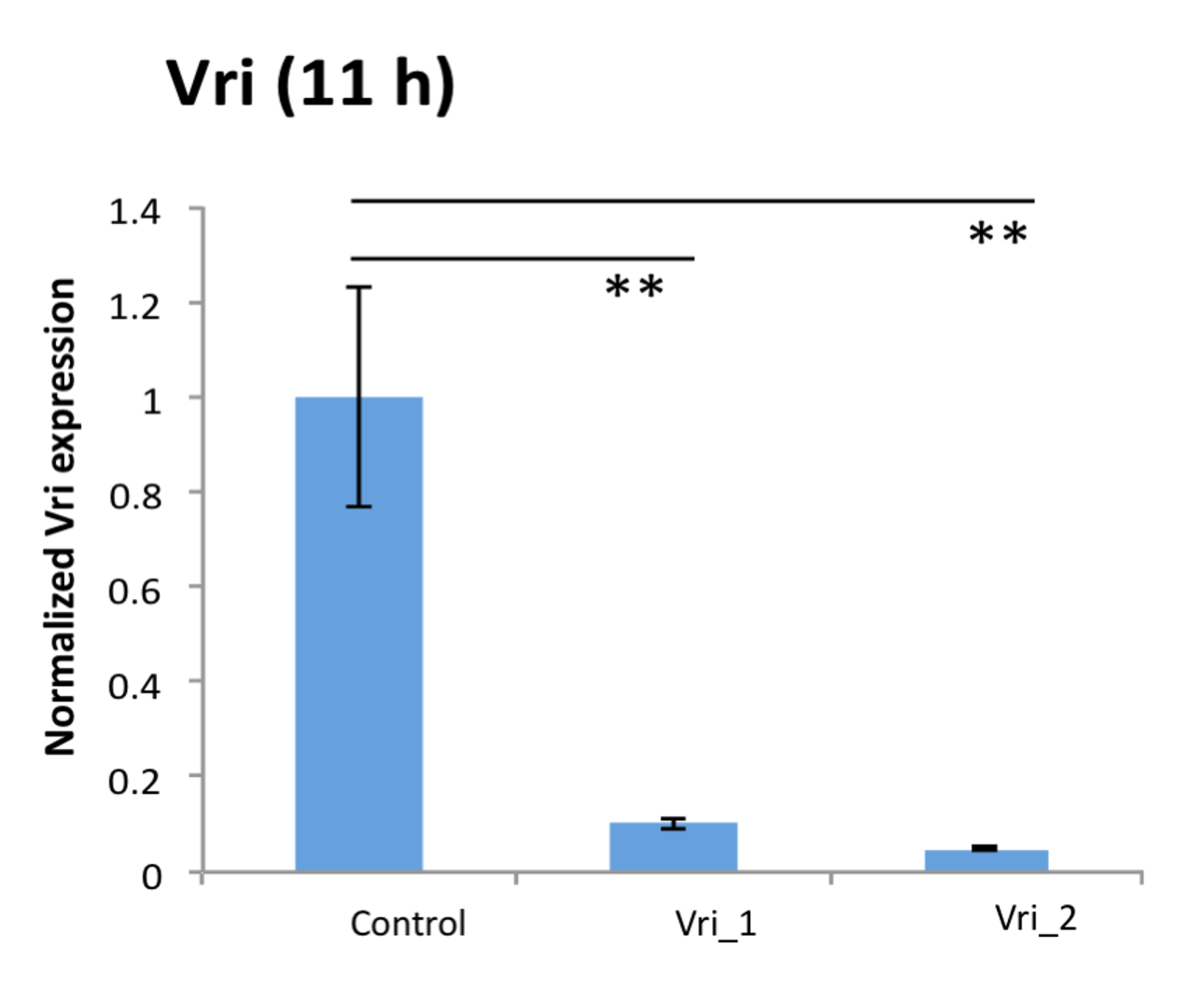

Supplement: S4 Fig — (Student's t-test; **, P<0.01). (TIFF) [file pgen.1006953.s004.tiff]

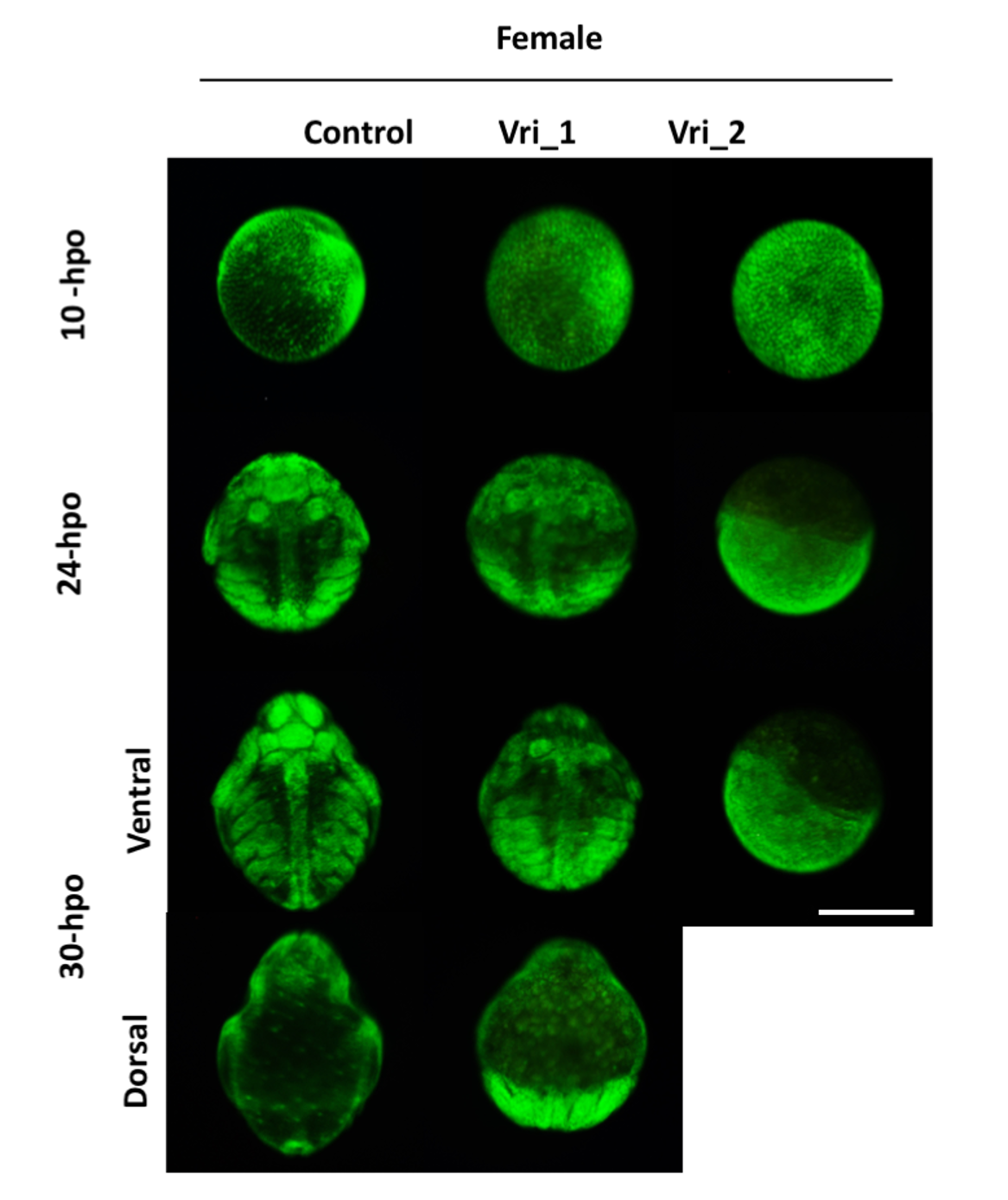

Supplement: S5 Fig — Scale bar: 200 μm. (TIFF) [file pgen.1006953.s005.tiff]

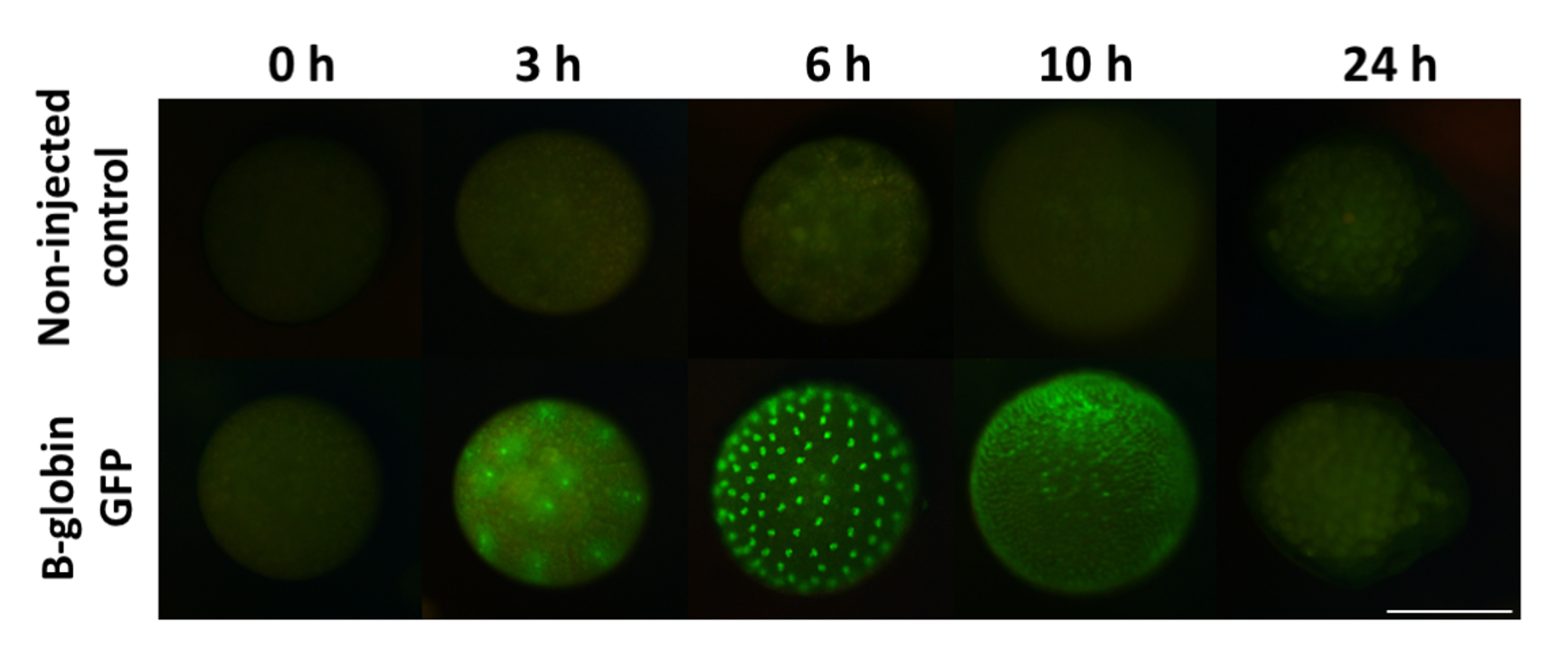

Supplement: S6 Fig — GFP was fused with Minos transposase, which resulted in nuclear localization of GFP as previously reported [54]. Scale bar: 200 μm. (TIFF) [file pgen.1006953.s006.tiff]

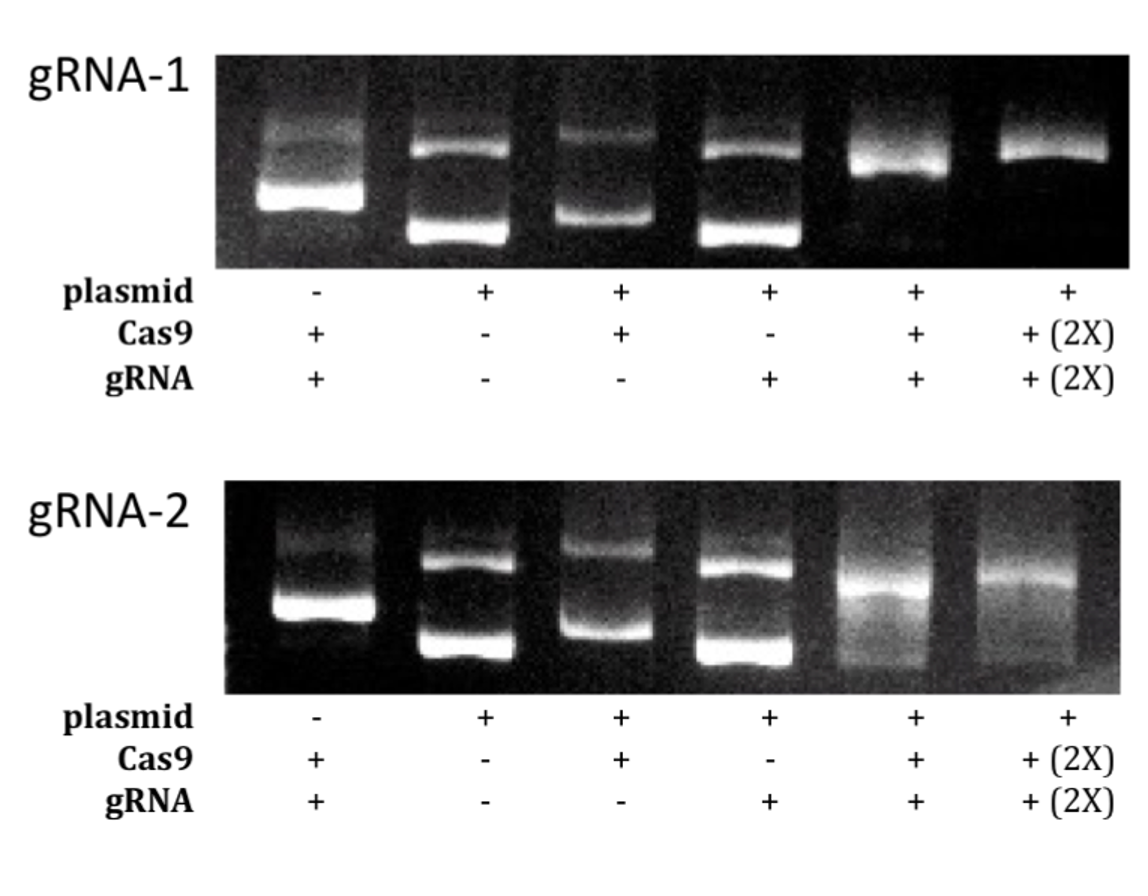

Supplement: S7 Fig — To examine the ability of designed gRNAs and Cas9 protein executing double strand break, 300 ng of plasmids harboring the target sequence were incubated with 1 μM Cas9 protein and 2 μM gRNA at 37°C for 1 hour in reaction buffer that contains 20 mM hepes (pH 7.5) 150 mM KCl, 0.5 mM DTT, 0.1 mM TCEP and 10 mM MgCl2. This reaction was stopped by adding 0.5 M EDTA to the reaction mixture. Cleavage of the plasmid by Cas9 and gRNA was observed by running the gel electrophoresis. The positive result can be interpreted by detecting the linear band of the plasmid on the gel. The target sequence for each gRNA (gRNA-1 and -2) is indicated in Fig 5. 2X means 2 μM Cas9 protein or 4 μM gRNA. (TIFF) [file pgen.1006953.s007.tiff]
