# Supplementary material for: Co-option of the bZIP transcription factor Vrille as the activator of Doublesex1 in environmental sex determination of the crustacean Daphnia magna
Source: PLoS Genet. 2017 Nov 2;13(11):e1006953. doi: 10.1371/journal.pgen.1006953 (PMC5667737; doi:10.1371/journal.pgen.1006953)
Supplement: S1 Table — (DOCX) [file pgen.1006953.s008.docx]

**S1 Table: Somatic mutagenesis in female for disrupting the *Dsx1* enhancer by CRISPR/Cas9 system.**

| Sex | Cas9 protein | gRNAs | Injected | Juvenile | Screened | Somatic mutation |
| --- | --- | --- | --- | --- | --- | --- |
| Female | 1 μM | 2 μM  each | 19 | 13 | 12 | 0 |
|  | 2.5 μM | 5 μM each | 45 | 11 | 11 | 0 |
|  | 2.5 μM | 2.5 μM each | 43 | 33 | 16 | 0 |
