# Supplementary material for: Co-option of the bZIP transcription factor Vrille as the activator of Doublesex1 in environmental sex determination of the crustacean Daphnia magna
Source: PLoS Genet. 2017 Nov 2;13(11):e1006953. doi: 10.1371/journal.pgen.1006953 (PMC5667737; doi:10.1371/journal.pgen.1006953)
Supplement: S2 Table — To evaluate heritable mutagenesis efficiency, we screened founder animals that produced progenies by genotyping. To feminize injected males, 100 μM Dsx1 siRNA was co-injected with Cas9 protein and gRNAs. (DOCX) [file pgen.1006953.s009.docx]

**S2 Table: Heritable mutagenesis in female and male for disrupting the *Dsx1* enhancer.**

| Sex | Cas9 | gRNAs | Injected | Juvenile | Adult | Screened | Mutated enhancer |
| --- | --- | --- | --- | --- | --- | --- | --- |
| Female | 1 uM | 2 uM each | 29 | 17 | 14 | 14 | 0 |
|  | 0.5 uM | 1 uM each | 13 | 7 | 5 | 5 | 0 |
| Male (coinjection with dsx1 siRNA) | 0.5 uM | 1 uM each | 66 | 11 | 8 | 8 | 0 |
|  | 1 μM | 1 μM  each | 66 | 4 | 3 | 3 | 0 |
|  | - | - | 22 | 15 | 15 | - | - |
