# Supplementary material for: Co-option of the bZIP transcription factor Vrille as the activator of Doublesex1 in environmental sex determination of the crustacean Daphnia magna
Source: PLoS Genet. 2017 Nov 2;13(11):e1006953. doi: 10.1371/journal.pgen.1006953 (PMC5667737; doi:10.1371/journal.pgen.1006953)
Supplement: S3 Table — (DOCX) [file pgen.1006953.s010.docx]

| **Gene name** | **Forward primer (5′-3′)** | **Reverse primer (5′-3′)** |
| --- | --- | --- |
| ***Vri*** | CATCCACATCACCAGCATCAC | CGCGACAACGACCAATCT |
| ***Dsx1*** | CCATTCATCATTACCAAATCCCTTC | CCATTCATCATTACCAAATCCCTTC |
| ***L32*** | GACCAAAGGGTATTGACAACAGA | CCAACTTTTGGCATAAGGTACTG |
| ***L8*** | GGTACTATTGTTTGCAATGTTGAGG | GTCTTCTTGGTATCGGTATTGTGAC |
| ***β-actin*** | TGTCTCTCTCTGTCCACGCTTTTC | TGTTGGGTGTCCTTGTGTGTC |
| ***Cyclophilin*** | GACTTTCCACCAGTGCCATT | AACTTTCCATCGCATCATCC |

**S3 Table: Primer sequences for Q-PCR**.
